# Applying behaviour change models to policy-making: development and validation of the Policymakers’ Information Use Questionnaire (POLIQ)

**DOI:** 10.1186/s12961-022-00942-y

**Published:** 2023-01-23

**Authors:** Keiko Shikako, Reem El Sherif, Roberta Cardoso, Hao Zhang, Jonathan Lai, Ebele R. I. Mogo, Tibor Schuster

**Affiliations:** 1 Faculty of Medicine and Health Sciences, School of Physical and Occupational Therapy, McGill University, Montréal, QC Canada; 2 Department of Family Medicine, McGill University, Montréal, QC Canada; 3 Research Institute, McGill University Health Center, Montréal, QC Canada; 4 Institute of Health Policy, Management and Evaluation, Dalla Lana School of Public Health, University of Toronto, Toronto, ON Canada; 5 Canadian Autism Spectrum Disorder Alliance (CASDA), Toronto, ON Canada; 6 Global Diet and Activity Research Group and Network, MRC Epidemiology Unit, University of Cambridge, Cambridge, UK; 7 MAB-Mackay Rehabilitation Centre, Montréal, QC Canada

**Keywords:** Questionnaire, Tool, Validation, Policy-makers, Behaviour change, Evidence-based

## Abstract

**Background:**

The purpose of this study was to develop and validate the Policymakers’ Information Use Questionnaire (POLIQ) to capture the intention of individuals in decision-making positions, such as health policy-makers, to act on research-based evidence in order to inform theory and the application of behaviour change models to decision-making spheres.

**Methods:**

The development and validation comprised three steps: item generation, qualitative face validation with cognitive debriefing and factorial construct validation. Confirmatory factor analysis was applied to estimate item–domain correlations for five predefined constructs relating to content, beliefs, behaviour, control and intent. Cronbach’s alpha coefficient was calculated to assess the overall consistency of questionnaire items with the predefined constructs. Participants in the item generation and face validation were health and policy researchers and two former decision-makers (former assistant deputy ministers) from the Canadian provincial level. Participants in the construct validation were 39 Canadian decision-makers at various positions of municipal, provincial and federal jurisdiction who participated in a series of policy dialogues focused on childhood disability.

**Results:**

Cognitive debriefing allowed for small adjustments in language for clarity, including simultaneous validation of the English and French questionnaires. Participants found that the questions were clear and addressed the domains being targeted. Internal consistency of items belonging to the respective questionnaire domains was moderate to high, with estimated Cronbach’s alpha values ranging from 0.67 to 0.84. Estimated item–domain correlations indicated moderate to high measurement performance for the domains norm, control and beliefs, whereas weak to moderate correlations resulted for the constructs content and intent. Estimated imprecision of factor loadings (95% confidence interval widths) was considerable for the questionnaire domains content and intent.

**Conclusion:**

Measuring decision-makers’ behaviour in relation to research evidence use is challenging. We provide initial evidence on face validity and appropriate measurement properties of the POLIQ based on a convenience sample of decision-makers in social and health policy. Larger validation studies and further psychometric property testing will support further utility of the POLIQ.

**Supplementary Information:**

The online version contains supplementary material available at 10.1186/s12961-022-00942-y.

## Background

The importance of the use of evidence-based knowledge in policy-making at the governmental and organizational levels has been well recognized, but there is evidence to suggest that translating this knowledge into practice is still a challenge [1]. Behaviour change models have been applied at the individual level of change, such as in testing drivers of the public’s adoption of, adherence to or perceptions of healthy behaviours; however, there is limited evidence on behavioural change from the perspective of decision-makers, who influence decisions that shape the macro context where health behaviours occur.

Despite a broad consensus that evidence should inform policy-making, it is known that the policy process is not linear and involves more than simple access to information. Similarly to other important behaviour changes, such as individual health behaviours and practitioners’ use of evidence-based information in clinical care, the use of academic evidence to guide health and social policy requires a combination of factors such as budgetary priorities, political climate, organizational culture and the presence of windows of opportunity [2, 3]. However, all these processes are initially triggered by individuals (hereafter called policy-makers), who must make a behavioural decision of accessing and using this information through these nonlinear processes of policy development. The conceptual use of knowledge implies changes in knowledge, understanding or attitudes. Research evidence use in health and social policy can include any of these approaches. Nevertheless, in all models, one must consider that research-based information could change thinking and inform decision-making but likely not determine the final course of behavioural action – the resulting policy [4].

Encouraging direct information exchanges and collaborative partnerships between researchers and policy-makers can be a key component of an effective knowledge translation (KT) strategy aiming at facilitating the uptake and increasing the use of evidence within policy decision-making contexts [5, 6]. To identify KT theories, models and frameworks (TMFs) that have the potential to maximize the impact of the research in patient care, services or policy change, a board of experts systematically reviewed 247 KT TMFs [7]. The experts used the criteria of usability, applicability and relevance of the TMF, and the outcome of the consensus exercise was to offer a menu approach so that researchers might select the TMF that was most suitable for the stage of KT they were working through. The menu includes three TMFs mapped onto the different parts of the KT process: the knowledge-to-action (KTA) framework for the knowledge creation process and KTA cycle [8] the EMTReK [9] for the transfer and exchange of knowledge, and the PRISM model [10] for implementation and sustainability and the Barwick planning template is recommended as an aid to planning [11]. There have been several models for conceptualizing evidence use by stakeholders [8, 12–16]. One of them is Larsen’s conceptual and instrumental knowledge use [17]. In this framework, conceptual knowledge use refers to using knowledge to change the way users think about issues. Instrumental knowledge use is the concrete application of knowledge and describes changes in behaviour that might, in the long run, change the final outcomes of evidence use in policy-making [17]. For example, a decision-maker who is constantly informed by research evidence may, at the moment that decisions must be made, access and use the information made available for him/her in the past. Weiss described several frameworks for knowledge use, including the problem-solving model, the direct application of the results of a study to a decision or using knowledge as “ammunition” to justify, consolidate or support a decision-making process [16].

Several studies have identified the need to better understand the use of evidence in policy-making [3, 18]. However, little is known about the individual-level behaviour changes necessary to systematically use evidence to inform the policy-making process. In a systematic literature review, Makkar et al. [19], found six tools developed to assess the utilization of research evidence by policy-makers. They highlighted the weaknesses of these tools, including not following a clear conceptual framework, assessing utilization of research evidence of a particular policy or in general, not considering the critical appraisal of research evidence, assessing the use of research evidence over a long period of time and hence increasing the possibility of recall bias, not using triangulation methods in data collection and, finally, not considering the imposed use of research evidence to meet organization or funding requirements for instance. To date, only one tool has been developed to measure the intention to use research evidence in policy-making, as reported by Boyko et al. [20]. Although the authors presented promising results in assessing the reliability of the tool, the study presented limitations including failure to assess the item–total correlation for each construct.

This study describes the development of the Policymakers’ Information Use Questionnaire (POLIQ), an instrument meant to assess behaviour change constructs for individuals in policy decision-making positions. The POLIQ was developed based on the theory of planned behaviour (TPB)’s constructs, namely, intention and the three constructs that feed into intention proximally—belief, norms and control. The use of these constructs is supported by several important conceptual frameworks in social and health psychology that suggest that behavioural intentions will result in behaviour changes [21].

In addition, the item–total correlation for each construct was assessed to determine whether items within each construct are sufficiently related to another [22]. Also, five aspects of intention, namely “likely to use”, “likely to share”, “intend to use”, “try to use” and “plan to use” were also included. This questionnaire aims to capture the intention of policy-makers (individuals in decision-making positions in government or organizations) to change behaviour by making research-based evidence-informed decisions, based on exposure to research information such as policy briefs and policy dialogues to cite a few. For the POLIQ, we operationalized policy briefs as a concise summary of research evidence on a particular issue, policy options to deal with it, and some recommendations based on the research evidence available [23]. Policy dialogues involve discussions among stakeholders to raise issues, share perspectives, find common ground and reach agreement or consensus, depending on the goal, on actionable solutions [24]. In this study, government policy-makers and others who were interested in formulating or influencing policy participated in a policy dialogue and received a policy brief on the issue.

The objective of this study is to describe the content development and face validity testing of the POLIQ.

## Methods

### Item generation

The initial version of the POLIQ was developed by combining key elements of the TPB Questionnaire [25, 26] and existing items from the Communicating Cancer Prevention (CCP) questionnaire for communication among state-level policy-makers ( [27]. The understanding that people change behaviour according to known steps and organized procedures has been used in implementation science and to inform behaviour interventions in a variety of fields [28]. The TPB posits that intention to perform a behaviour is best predicted when individuals have a positive evaluation of the behaviour (attitudes), believe peers will support the behaviour (subjective norm) and perceive the behaviour to be within their capabilities (perceived behavioural control, PBC) [29]. It also aligns with Roger’s diffusion of innovations theory, which posits that people will disseminate newly acquired knowledge when it resonates with their current needs, when they are in a position to affect change with this information, and when their cultural and ethical values align with the innovation being spread [30].

**Fig. 1:**
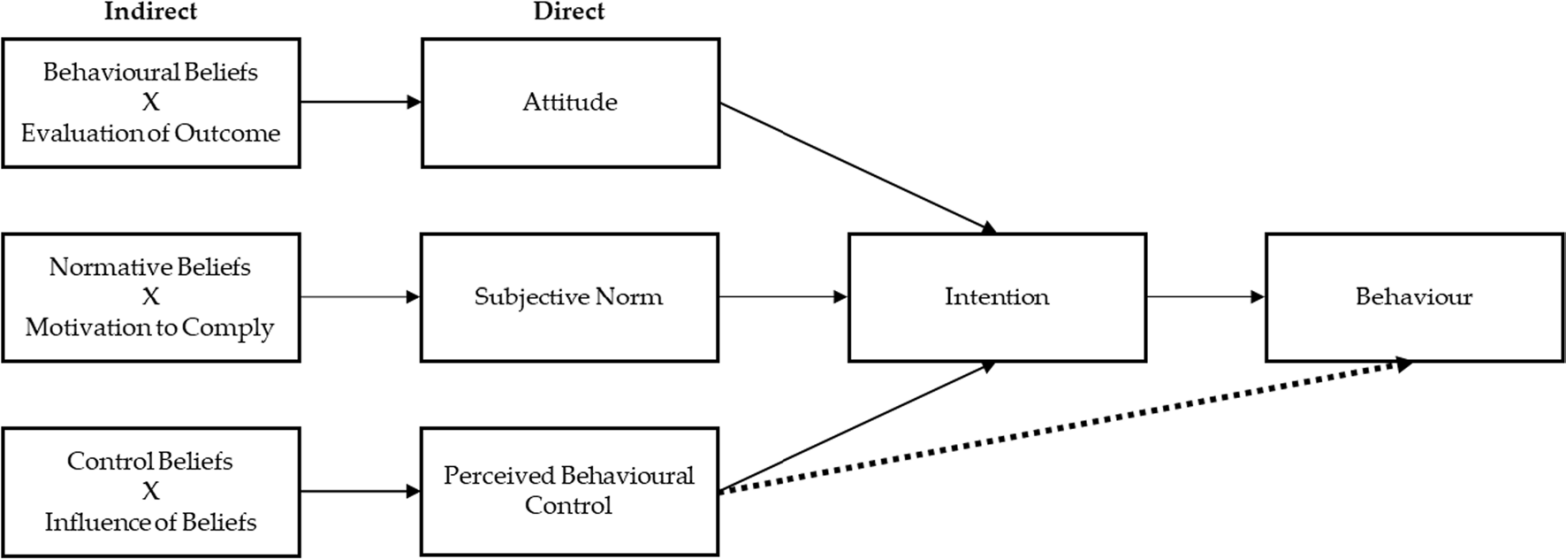
The theory of planned behaviour [19]

While TPB has often been used in behaviour change research and designing interventions [25, 26], it has rarely been applied to the field of informing policy. TPB factors can be assessed directly (e.g. by asking people to report attitudes, norms and PBC) or indirectly (e.g. by asking people about specific behavioural beliefs and combining the scores with a paired evaluation of the belief) (see Fig. 1). To adapt it to the goals of this project, the TPB’s constructs were mapped to each of the TPB constructs, namely, intention and the three constructs that feed into intention proximally—belief, norms and control. The focus of the questions was changed so that they referred to the policy briefs provided at the policy dialogue (e.g. where the original question referred to control factors relating to exercise, the questions were changed to control factors relating to policy influence such as resources, relationships and organizational support); and the focus of the normative questions was changed to concern the perception of professional peers rather than the perceptions of friends and family given the professional context of the dialogues.

The CCP was designed to examine responses of policy-makers to policy briefs [27]. To modify its original items, we changed the introductory paragraphs and survey questions to make them refer to the policy briefs provided at the policy dialogue related to different areas of childhood disability research instead of cancer prevention; removed the open-ended aspects of this survey and only included the Likert scale questions; eliminated questions focused on participants demographic information such as political leaning, income level, family information and health status; and added a question to capture stakeholders’ preferred brief format.

**Fig. 2:**
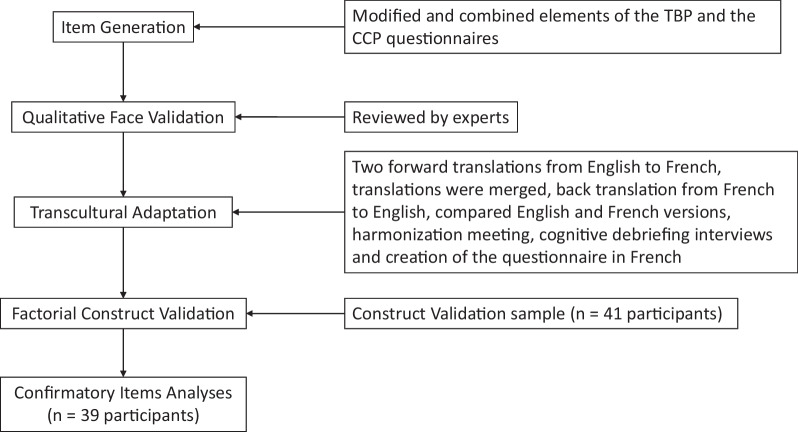
Flow chart of Policymakers’ Information Use Questionnaire development

Then, all modified questions were merged, without associating them to the related constructs, and the POLIQ was created. The following steps were taken to further develop and validate the questionnaire (Fig. 2).

### Qualitative face validation

The first step of face validation involved reviewing the questionnaire with a convenience purposeful sample of researchers participating in a health policy-related conference, in the first week of November 2017. A policy-maker (who has also served as an assistant deputy minister in Ontario, Canada) as well as a social work professor from a Canadian university were consulted. While they generally found the length and content reasonable, there was discomfort with the wording of some questions that elicited emotional responses (e.g. do you feel you can use this information to advance personal causes) or were perceived as leading questions. These questions were removed or reworded. We were also advised by these expert reviewers to harmonize the Likert scale labels for each question to avoid confusion. A third researcher in a different field of health policy (global health governance) was also invited to participate in the cognitive debriefing of the revised version with the new Likert scale labels and found that the items were clear for the intended purpose.

### Transcultural adaptation

The questionnaire was developed in English and translated into French to account for the two official languages in Canada. To that end, a rapid rigorous transcultural adaptation of the questionnaire was performed following methodological guidance developed and previously validated by members of this team [31]. The following steps were performed: (1) two bilingual translators conducted a forward translation from English to French, (2) the two French versions were merged into one version, (3) one bilingual translator conducted a back translation of the merged version into English, (4) the back-translated questionnaire was compared with the original version for discrepancies to be highlighted and resolved, (5) a harmonization meeting was held with the bilingual team members to agree on a harmonized version of the questionnaire in French, (6) two cognitive debriefing interviews with native French speakers were conducted and feedback was collected on the questionnaire items, and (7) a final team meeting was held to review this feedback and agree on the final version of the French version of the questionnaire.

### Data collection

During three policy dialogues held in three Canadian provinces (Quebec, Ottawa and British Columbia), 52 policy attendees were invited to respond to the questionnaire with a total enrolment of 41 participants. The policy dialogues were events led by members of this team with the objective of informing policy-makers in health and social policy positions about different areas of childhood disability research. The first policy dialogue was conducted in British Columbia, focused on research informing the inclusion of children with disabilities in leisure opportunities with provincial and municipal-level government decision-makers, and leaders of large provincial nongovernmental organizations (NGOs). The second policy dialogue was conducted in Ontario, focused on research on rights-based approaches in childhood disabilities, and participants were provincial and federal bureaucracy members (disability issues and children and family ministries’ representatives). The third policy dialogue was held in Quebec and focused on research-based strategies to include children with disabilities in summer camps and community activities, and participants were provincial and municipal elected officials, bureaucracy and NGO leaders.

The detailed methodology for the policy dialogues is described elsewhere [32, 33]. In all three dialogues, attendees received a policy brief 2 weeks prior to the event, in their preferred language (English or French). Participants engaged in a 3-h meeting with passive (lecture) and active (facilitated small work group discussions with focused questions and requested feedback and application to the large group) learning and exchange opportunities. Participants were given time to complete the POLIQ immediately after the meeting or asked to complete it within 2 weeks and return it by mail or email, according to their preference. 

### Factorial construct validation

The POLIQ is composed of questionnaire items that aim to map three latent domains which lead to the intention to act: beliefs, norms and control. Each of the three domains comprises complementary sub-domains: beliefs (behaviour and attitude towards beliefs), norms (beliefs and subjective view of those normative beliefs) and control (beliefs and perception of control beliefs). In addition, there are five items that measure aspects of intention, namely “likely to use”, “likely to share”, “intend to use”, “try to use” and “plan to use”. Each questionnaire item measures agreement with the respective item statement on a five-point Likert scale (1 strongly disagree; 2 disagree, 3 neutral, 4 agree and 5 strongly agree). To reflect a possible lack of applicability of a question, a sixth category “not applicable” was added. An additional file shows the questionnaire, the items and respective domains [see Additional file 1].

Confirmatory factor analysis was applied in order to estimate item–domain correlations (factor loadings) for items in each of the five predefined domains. Cronbach’s alpha coefficient was calculated to assess the overall consistency of questionnaire items within the five predefined latent constructs of the questionnaire. Due to the limited sample size, the robustness of results was assessed using bootstrap sampling and 95% bootstrap confidence intervals were provided for item–domain correlations and Cronbach’s alpha estimates.

**Fig. 3:**
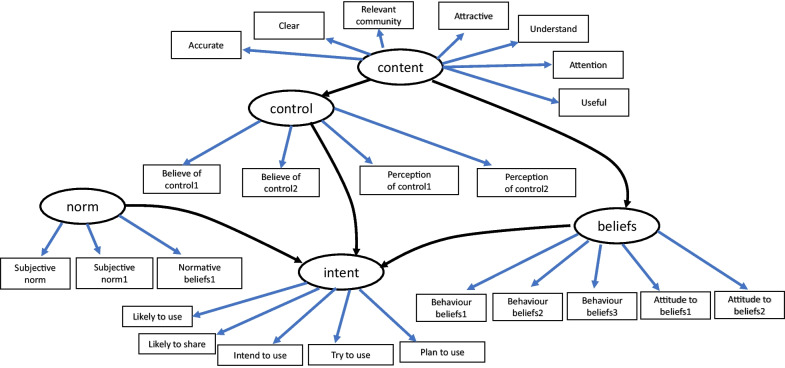
Conceptional item–domain (arrow in black ink) and inter-domain (arrow in blue ink) dependencies for the Policymakers’ Information Use Questionnaire (POLIQ). Legend: Strongly disagree, Disagree, Neutral, Agree, Strongly agree, N/A

Statistical analyses were performed using the R software [34]. The Lavaan package was used to perform confirmatory factor analysis and based on the fitted structural model, standardized factor loadings were computed to estimate item–domain correlations. Based on the underlying theoretical model (Fig. 3), independence constraints were imposed between the items of the three domains: beliefs, control and norms.

## Results

Because of incomplete questionnaire responses obtained from two individuals, all statistical analyses were performed using the complete case set of 39 individuals.

### Item response distribution

**Fig. 4:**
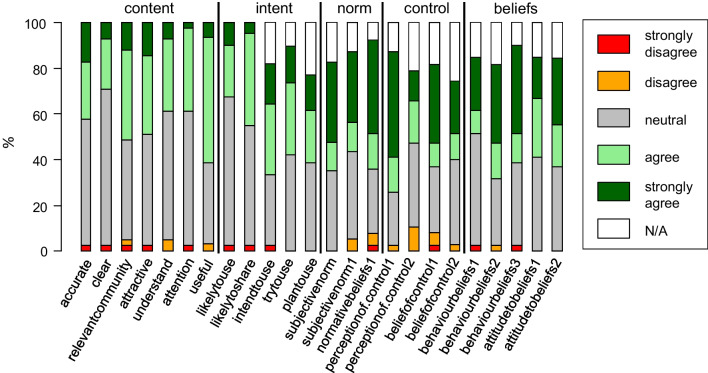
Response distribution Policymakers’ Information Use Questionnaire (POLIQ), sample size *n* = 39

Figure 4 displays the relative frequencies of responses across the 24 questionnaire items. For all questionnaire items, the relative frequency of strong disagreement and/or disagreement was low (< 10% for 22/24 items). Otherwise, moderate heterogeneity and fair prevalence of item responses were observed across all items of the questionnaire domains norm, control and beliefs. One item (“likely to share”) in the intent domain and two items in the content domain (“understand”, “attention”, “useful”) indicated low prevalence of strong agreement (relative frequencies < 10%). With a range of 28–66%, the prevalence of neutral responses was relatively high across all items. The prevalence of responses indicating a lack of applicability (“N/A”) ranged from 0 to 23% across the domains norm, control, beliefs and intent.

### Internal consistency and item–domain correlations

**Fig. 5:**
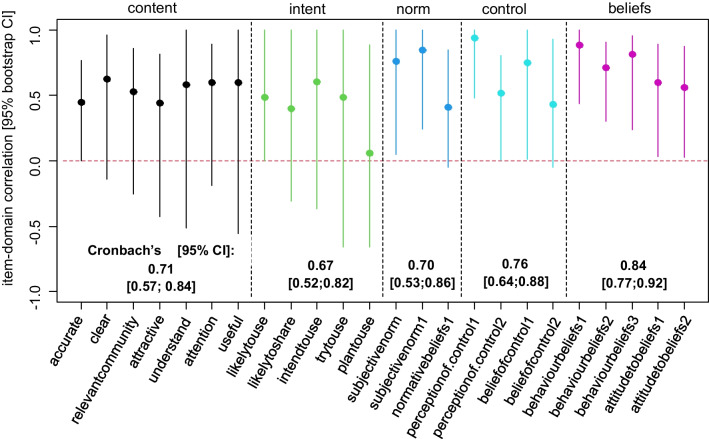
Estimated item–domain correlations and internal consistency indices (Cronbach’s alpha) for the Policymakers’ Information Use Questionnaire (POLIQ), sample size *n* = 39

Overall internal consistency of the questionnaire was moderate to high based on the sample data as indicated by a range of Cronbach’s alpha values of 0.67–0.84 for the five latent constructs. Overall, moderate to high item–domain correlations were found for the questionnaire items within the five domains based on the study data (Fig. 5). However, imprecision of correlation estimates was high for items belonging to the questionnaire constructs “content” and “intent”, precluding a conclusive assessment.

**Fig. 6:**
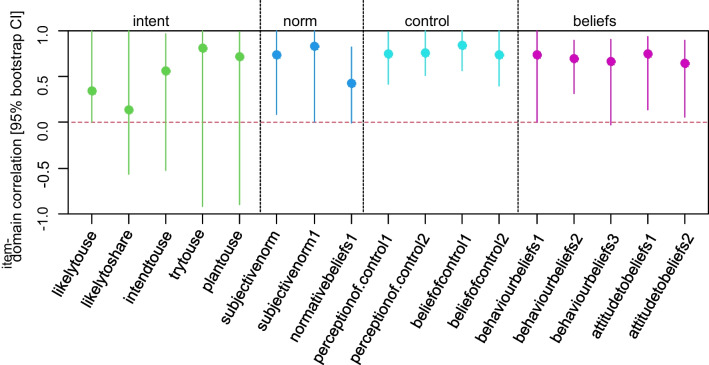
Estimated item–domain correlations for the Policymakers’ Information Use Questionnaire (POLIQ), sample size *n* = 39 [construct “content’ removed from the structural model for purpose of sensitivity analysis]

The results of the confirmatory factor analysis were consistent with the findings of a sensitivity analysis that excluded the construct “content” that embodied the largest number of items and was theoretically considered to be predictive for the remaining questionnaire constructs (Fig. 6).

## Discussion

The TPB model proposes three constructs that drive behaviour: attitudes, subjective norms and PBC [25, 26]. Two of those constructs (subjective norm and PBC) are not modifiable in terms of KT and implementation science efforts, whereas attitudes towards the information received may be modulated through active implementation strategies [25]. Complementary to Brownson’s work [27], in this study we offer an additional construct of “content” in understanding the intention of policy-makers to act on research information. We added this construct based on the research suggesting that evidence use in policy-making is highly specific to the perceived applicability of the content, the roles attributed to the individual policy-maker and the content fit to the policy agendas [26]. One use of this tool is to support KT science in expanding the use of the KTA framework from the health practice to the health policy-making sphere [8]. The KTA framework posits that targeted KT strategies can be adopted to support the use of evidence, and therefore, it becomes paramount to measure constructs that can be modified—for example, elements of intention to act such as *content*—that we propose to measure with the POLIQ.

The distribution of the questionnaire responses indicated a low prevalence of disagreement or strong disagreement across all items. As face validity of the questionnaire was established before deploying the sample survey, we assume that this skewed pattern of answers is due to the selection of survey participants. Participants in the validation study were attendees of policy dialogues in childhood disabilities and were therefore actively engaged with the research content provided and to which they responded in the questionnaire. It is unlikely that improper wording of the questionnaire items made it “difficult to disagree” and more likely that the purposeful sampling strategy and the KT strategies used contributed to a higher agreement with pertinence and utility of the research evidence. Our sample consisted of participants who had a history of engaging with research and in particular with the researchers leading the activities, which may have favoured participation and engagement. However, this close relationship and the noncontentious nature of the topic (i.e. no policy-maker represented would say that promotion of leisure or human rights for children with disabilities is not an important topic) may have also led to the relatively large proportion of respondents who neither agreed nor disagreed with statements in the questionnaire, indicating hesitation to commit to a definite decision, that is, may indicate a social desirability response bias or “diplomatic response behaviour” previously observed among policy-makers [27, 36].

Our results, moderate (+0.3 < *r* < +0.5) to high (*r* ≥ +0.5) item–domain correlations for the constructs norm, beliefs and behaviour, are consistent with other studies using the TPB model. In the original TPB we see that the beliefs (*r* = +0.40), control (*r* = +0.26) and norms (*r* = +0.28) were all associated with intent. This is what we expected and is in line with previous literature using the TPB. When we removed the construct content from the structural model, we observed expected change in the correlation structure: as content was strongly associated with intent (*r* = +0.52), the associations of the constructs control, beliefs and norms increased once content was removed from the model. In the saturated model, content was not associated with beliefs (*r* = +0.03). Interestingly, content was negatively associated with control (*r* = −0.29) and norms (*r* = −0.44). These correlations were not surprising as they confirm the perception that policy content should not be associated with the policy-makers’ individual beliefs and values, but rather respond to collective needs and political agendas [37].

Different tools exist to support the assessment of evidence use by decision-makers [7]. However, to date, there are only two questionnaires, including the POLIQ, that aim to measure the intention to use research evidence in policy-making [20]. The POLIQ was developed to address the limitations that we had identified in using another tool [27], and expands on the measurement of intention by including the TPB’s constructs that feed into the intention construct proximally: belief, norms and control. We expect to advance the understanding of how policy-makers use research evidence following KT strategies, such as access to policy briefs or interactive policy dialogues with researchers and other stakeholders. We propose to assess this through four aspects of intention, which were moderately to highly correlated with the intention construct in the POLIQ pilot testing: likelihood to use and share information, intention to use, tentative to sue and plan to use the information. The added items related specifically to content received are also important, as we know the value of specificity and perceived context utility of information for decision-making processes [38].

### Study limitations

Due to the limited sample size and the relatively large number of estimated parameters of the structural model, the precision of estimates obtained was low to fair. However, parameter estimates yielded (with one exception) moderate to high item–domain correlations, indicating consistency with desired measurement properties of the proposed instrument.

The prevalence of neutral responses was relatively high across all items and domains. We believe this is also due to the limited sample size, and the fact that different policy-makers attending the events and responding to the questionnaire, were interested in specific items or have roles in conveying information to others (e.g. policy analysts), using the information to advocate for their constituency (e.g. NGO leaders) and not making decisions themselves. The consideration of the target population to receive specific information is fundamental when developing KT strategies to inform policy-making. The POLIQ would better suit as a measure of intention to use research evidence following KT strategies done with an audience of individuals at the government level who inform the policy process such as bureaucracy directly selecting evidence to inform the leadership about policy options.

The study findings provide initial evidence on face validity and appropriate measurement properties of the POLIQ based on a convenience sample of health and social policy-makers. Larger consecutive validation studies in relevant populations are needed to further establish the utility and further psychometric characteristics of the POLIQ.

## Conclusion

Implementation of research-based evidence into complex systems such as public health and social policy requires an active development of partnerships across stakeholders in research and policy-making, and a deep understanding of the human behaviours involved in the process. The use of the theory of behaviour change to guide implementation can support the development of better-targeted and more effective strategies. A standardized questionnaire that accounts for beliefs, control and norms, and content of the research-based evidence, applied to the context of policy-making, can support a better understanding about how individuals in decision-making positions intend to use information provided to them. This knowledge may provide insights into implementation and research-evidence use in policy-making. Researchers can learn to generate evidence that informs policy and to develop targeted strategies to support sustaining behavioural change in policy-makers towards accepting, disseminating and using research-based evidence.

## Supplementary Information

**Additional file 1.** The POLIQ tool—the questionnaire, the items and respective domains.

## Data Availability

Data are available from the authors.

## Abbreviations

POLIQ: Policymakers’ Information Use Questionnaire
TPB: Theory of planned behaviour
CCP: Communicating Cancer Prevention Questionnaire
PBC: Perceived behavioural control
