# Supplementary material for: Applying behaviour change models to policy-making: development and validation of the Policymakers’ Information Use Questionnaire (POLIQ)
Source: Health Res Policy Syst. 2023 Jan 23;21:8. doi: 10.1186/s12961-022-00942-y (PMC9872298; doi:10.1186/s12961-022-00942-y)
Supplement: Supplementary file 1 — Additional file 1. The POLIQ tool—the questionnaire, the items and respective domains. [file 12961_2022_942_MOESM1_ESM.pdf]

#	Variable / Field Name	Field Label<br>***Field Note***	Field Attributes (Field Type, Validation, Choices, Calculations, etc.)
Instrument: **post-dialogues_2wk** (postdialogues_2wk)			
1	record_id	Study ID	text, Identifier
2	participant_id	Participant ID	text, Identifier
3	participant_name2wks	Please enter your first and last name:	text, Identifier
4	participant_org_2wks	What is the name of your organization?	text, Identifier
5	participant_position_2wks	What is your job title/position in your organization?	text, Identifier
6	brief_preference	Which of the briefs did you find most useful/valuable?	slider<br>Slider labels: The research based brief, , The narrative based brief<br>Custom alignment: LH
7	content_amountread	How much of the preferred brief did you read?	radio<br>0 Almost none of it<br>1 Some of it<br>2 Most of it<br>3 All of it<br>Custom alignment: LH
8	content_closelyread	How closely did you read the preferred brief?	radio<br>0 Not at all closely<br>1 Somewhat closely<br>2 Very closely<br>Custom alignment: LH
9	content_believe	*The following questions are made of a series of statements. Please indicate the extent to which you agree with each of them by selecting the appropriate choice. (Adapted from Communicating Cancer Prevention among State-Level Policy Makers in Brownson et al. (2011). Communicating evidence-based information on cancer prevention to state-level policy makers. Journal of the National Cancer Institute, 103(4), 306-316.)*<br>The information in the policy briefs is believable.	radio (Matrix)<br>0 Strongly disagree<br>1 Disagree<br>2 Agree<br>3 Strongly agree<br>4 Neither agree nor disagree<br>5 (SKIP)<br>6 Not applicable
10	content_accurate	The information in the policy briefs is accurate.	radio (Matrix)<br>0 Strongly disagree<br>1 Disagree<br>2 Agree<br>3 Strongly agree<br>4 Neither agree nor disagree<br>5 (SKIP)<br>6 Not applicable
11	content_relevantlife	The information in the policy briefs is relevant to my job.	radio (Matrix)<br>0 Strongly disagree<br>1 Disagree<br>2 Agree<br>3 Strongly agree<br>4 Neither agree nor disagree<br>5 (SKIP)<br>6 Not applicable
12	content_clear	The information in the policy briefs is clear.	radio (Matrix)<br>0 Strongly disagree<br>1 Disagree<br>2 Agree<br>3 Strongly agree<br>4 Neither agree nor disagree<br>5 (SKIP)<br>6 Not applicable
13	content_relevantcommunity	The information in the policy briefs is relevant to my mandate/stakeholders.	radio (Matrix)<br>0 Strongly disagree<br>1 Disagree<br>2 Agree<br>3 Strongly agree<br>4 Neither agree nor disagree<br>5 (SKIP)<br>6 Not applicable

14	content_interesting	The information in the policy briefs is interesting.	radio (Matrix)<br>0 Strongly disagree<br>1 Disagree<br>2 Agree<br>3 Strongly agree<br>4 Neither agree nor disagree<br>5 (SKIP)<br>6 Not applicable
15	content_attractive	The information in the policy briefs is presented in an attractive way.	radio (Matrix)<br>0 Strongly disagree<br>1 Disagree<br>2 Agree<br>3 Strongly agree<br>4 Neither agree nor disagree<br>5 (SKIP)<br>6 Not applicable
16	content_wllingness	The information increased my willingness to influence policy for children with disabilities.	radio (Matrix)<br>0 Strongly disagree<br>1 Disagree<br>2 Agree<br>3 Strongly agree<br>4 Neither agree nor disagree<br>5 (SKIP)<br>6 Not applicable
17	content_emotion	The information in the policy briefs affected me emotionally.	radio (Matrix)<br>0 Strongly disagree<br>1 Disagree<br>2 Agree<br>3 Strongly agree<br>4 Neither agree nor disagree<br>5 (SKIP)<br>6 Not applicable
18	content_understand	The information in the policy briefs is easy to understand.	radio (Matrix)<br>0 Strongly disagree<br>1 Disagree<br>2 Agree<br>3 Strongly agree<br>4 Neither agree nor disagree<br>5 (SKIP)<br>6 Not applicable
19	content_attention	The information in the policy briefs held my attention.	radio (Matrix)<br>0 Strongly disagree<br>1 Disagree<br>2 Agree<br>3 Strongly agree<br>4 Neither agree nor disagree<br>5 (SKIP)<br>6 Not applicable
20	content_importantissue	The information in the policy briefs helps me relate to an important issue facing my stakeholders.	radio (Matrix)<br>0 Strongly disagree<br>1 Disagree<br>2 Agree<br>3 Strongly agree<br>4 Neither agree nor disagree<br>5 (SKIP)<br>6 Not applicable
21	content_useful	I am likely to use the information in this brief.	radio (Matrix)<br>0 Strongly disagree<br>1 Disagree<br>2 Agree<br>3 Strongly agree<br>4 Neither agree nor disagree<br>5 (SKIP)<br>6 Not applicable
22	content_shareable	I am likely to share the information in this brief with a colleague.	radio (Matrix)<br>0 Strongly disagree<br>1 Disagree<br>2 Agree<br>3 Strongly agree<br>4 Neither agree nor disagree<br>5 (SKIP)<br>6 Not applicable

23	content2_believe	The information in the policy briefs is believable	radio (Matrix)<br>0 Strongly disagree<br>1 Disagree<br>2 Agree<br>3 Strongly agree<br>4 Neither agree nor disagree<br>5 (SKIP)<br>6 Not applicable
24	content2_accurate	The information in the policy briefs is accurate	radio (Matrix)<br>0 Strongly disagree<br>1 Disagree<br>2 Agree<br>3 Strongly agree<br>4 Neither agree nor disagree<br>5 (SKIP)<br>6 Not applicable
25	content2_relevant	The information in the policy briefs is relevant to my job	radio (Matrix)<br>0 Strongly disagree<br>1 Disagree<br>2 Agree<br>3 Strongly agree<br>4 Neither agree nor disagree<br>5 (SKIP)<br>6 Not applicable
26	content2_clear	The information in the policy briefs is clear	radio (Matrix)<br>0 Strongly disagree<br>1 Disagree<br>2 Agree<br>3 Strongly agree<br>4 Neither agree nor disagree<br>5 (SKIP)<br>6 Not applicable
27	content2_relevant_stake	The information in the policy briefs is relevant to my mandate/stakeholders	radio (Matrix)<br>0 Strongly disagree<br>1 Disagree<br>2 Agree<br>3 Strongly agree<br>4 Neither agree nor disagree<br>5 (SKIP)<br>6 Not applicable
28	content2_interesting	The information in the policy briefs is interesting	radio (Matrix)<br>0 Strongly disagree<br>1 Disagree<br>2 Agree<br>3 Strongly agree<br>4 Neither agree nor disagree<br>5 (SKIP)<br>6 Not applicable
29	content2_attractive	The information in the policy briefs is presented in an attractive way	radio (Matrix)<br>0 Strongly disagree<br>1 Disagree<br>2 Agree<br>3 Strongly agree<br>4 Neither agree nor disagree<br>5 (SKIP)<br>6 Not applicable
30	content2_willingness	The information increased my willingness to influence policy for children with disabilities	radio (Matrix)<br>0 Strongly disagree<br>1 Disagree<br>2 Agree<br>3 Strongly agree<br>4 Neither agree nor disagree<br>5 (SKIP)<br>6 Not applicable
31	content2_understandable	The information in the policy briefs is easy to understand	radio (Matrix)<br>0 Strongly disagree<br>1 Disagree<br>2 Agree<br>3 Strongly agree<br>4 Neither agree nor disagree<br>5 (SKIP)<br>6 Not applicable

32	content2_attention	The information in the policy briefs held my attention	radio (Matrix)<br>0 Strongly disagree<br>1 Disagree<br>2 Agree<br>3 Strongly agree<br>4 Neither agree nor disagree<br>5 (SKIP)<br>6 Not applicable
33	content2_issues	The information in the policy briefs helps me relate to an important issue facing my stakeholders	radio (Matrix)<br>0 Strongly disagree<br>1 Disagree<br>2 Agree<br>3 Strongly agree<br>4 Neither agree nor disagree<br>5 (SKIP)<br>6 Not applicable
34	content2_useful	I am likely to use the information in the briefs	radio (Matrix)<br>0 Strongly disagree<br>1 Disagree<br>2 Agree<br>3 Strongly agree<br>4 Neither agree nor disagree<br>5 (SKIP)<br>6 Not applicable
35	content2_share	I am likely to share the information in the briefs with a colleague	radio (Matrix)<br>0 Strongly disagree<br>1 Disagree<br>2 Agree<br>3 Strongly agree<br>4 Neither agree nor disagree<br>5 (SKIP)<br>6 Not applicable
36	intent1_likelytousr	Section Header: *The following questions are related to the information you gained through the briefs and the policy dialogue. (Adapted from Constructing A Theory Of Planned Behavior Questionnaire. (2017). TPB Questionnaire Construction. Retrieved 3 October 2017, from http://people.umass.edu/aizen/pdf/tpb.measurement.pdf)*<br>I am likely to use the information in the brief	radio<br>0 strongly disagree<br>1 disagree<br>2 agree<br>3 strongly agree<br>4 neither<br>5 skip<br>6 NA
37	intent1_likelytoshare	I am likely to share the information in the brief with a colleague	radio<br>0 strongly disagree<br>1 disagree<br>2 agree<br>3 strongly agree<br>4 neither<br>5 skip<br>6 NA
38	intent1_intendtouse	I intend to use the information I gained to influence policy for children with disabilities in the next 12 months.	radio<br>0 strongly disagree<br>1 disagree<br>2 agree<br>3 strongly agree<br>4 neither<br>5 skip<br>6 NA
39	beliefs_behav1	If I use the information I gained to influence policy, my action will result in their improved participation in leisure and physical activity.	radio<br>0 strongly disagree<br>1 disagree<br>2 agree<br>3 strongly agree<br>4 neither<br>5 skip<br>6 NA
40	norm_subj2	Colleagues whose opinions I value would influence policy with the information I gained through the briefs and the policy dialogue.	radio<br>0 strongly disagree<br>1 disagree<br>2 agree<br>3 strongly agree<br>4 neither<br>5 skip<br>6 NA

41	intent1_trytouse	I will try to use the information I gained to influence policy for children with disabilities in the next 12 months.	radio<br>0 strongly disagree<br>1 disagree<br>2 agree<br>3 strongly agree<br>4 neither<br>5 skip<br>6 NA
42	beliefs_behav2	My professional influence on policy using the information gained will likely result in the improved participation of children with disabilities in leisure and physical activity.	radio<br>0 strongly disagree<br>1 disagree<br>2 agree<br>3 strongly agree<br>4 neither<br>5 skip<br>6 NA
43	control_belief1	I expect that I will have the organizational resources to influence policy on children with disabilities in the next 12 months.	radio<br>0 strongly disagree<br>1 disagree<br>2 agree<br>3 strongly agree<br>4 neither<br>5 skip<br>6 NA
44	intent1_plantouse	I plan to use the information I gained to influence policy for children with disabilities in the next 12 months.	radio<br>0 strongly disagree<br>1 disagree<br>2 agree<br>3 strongly agree<br>4 neither<br>5 skip<br>6 NA
45	beliefs_behav3	My organization's influence on policy using the information gained will result in the improved participation of children with disabilities in leisure and physical activity.	radio<br>0 strongly disagree<br>1 disagree<br>2 agree<br>3 strongly agree<br>4 neither<br>5 skip<br>6 NA
46	control_belief2	Having the organizational resources to influence policy on children with disabilities in the next 12 months will enable me to do so.	radio<br>0 strongly disagree<br>1 disagree<br>2 agree<br>3 strongly agree<br>4 neither<br>5 skip<br>6 NA
47	beliefs_attitude1	To use this information to influence policy for children with disabilities in the next 12 months will be personally fulfilling.	radio<br>0 strongly disagree<br>1 disagree<br>2 agree<br>3 strongly agree<br>4 neither<br>5 skip<br>6 NA
48	norm_beliefs1	Most people in my field would use the information gained to influence policy for children.	radio<br>0 strongly disagree<br>1 disagree<br>2 agree<br>3 strongly agree<br>4 neither<br>5 skip<br>6 NA
49	control_perception1	I am confident that I can influence policy on children with disabilities in the next 12 months.	radio<br>0 strongly disagree<br>1 disagree<br>2 agree<br>3 strongly agree<br>4 neither<br>5 skip<br>6 NA

50	beliefs_attitude2	To use this information to influence policy for children with disabilities in the next 12 months will be impactful for me.	radio<br>0 strongly disagree<br>1 disagree<br>2 agree<br>3 strongly agree<br>4 neither<br>5 skip<br>6 NA
51	norm_subj1	Colleagues I respect would try to use the information I gained through the briefs and the policy dialogue to influence policy.	radio<br>0 strongly disagree<br>1 disagree<br>2 agree<br>3 strongly agree<br>4 neither<br>5 skip<br>6 NA
52	control_perception2	Influencing policy on children with disabilities in the next 12 months is up to me.	radio<br>0 strongly disagree<br>1 disagree<br>2 agree<br>3 strongly agree<br>4 neither<br>5 skip<br>6 NA
53	moreinformed	As a result of my participation in this activity, I am better informed about rights-based approaches in childhood disabilities	radio<br>0 strongly disagree<br>1 disagree<br>2 agree<br>3 strongly agree<br>4 neither<br>5 skip<br>6 NA
54	overall_satisfy	Overall I was satisfied	radio<br>0 strongly disagree<br>1 disagree<br>2 agree<br>3 strongly agree<br>4 neither<br>5 skip<br>6 NA
55	overall_timeuse	This activity was a good use of my time	radio<br>0 strongly disagree<br>1 disagree<br>2 agree<br>3 strongly agree<br>4 neither<br>5 skip<br>6 NA
56	bestthing_pd	What was best thing about this PD?	notes
57	improvment	ID at least one improvement we could make for future engagement activities	notes
58	other_comments	additional comments?	notes
59	postdialogues_2wk_complete	Section Header: *Form Status*<br>Complete?	dropdown<br>0 Incomplete<br>1 Unverified<br>2 Complete
Instrument: **post-dialogues_6mo** (postdialogues_6mo)			
60	instructions_v2	It has been six months since you participated in the BC Policy Dialogue in childhood disabilities. After the session, we sent you a questionnaire on your perception of the briefs in terms of content, format, and utility.<br><br>Now we would like to understand if the information that you received during the Dialogue and through the briefs was used somehow or applied in any way, and if not, the reasons why.<br><br>All information you provide will be anonymous and confidential.<br><br>This survey [1] should take only ten minutes of your time.<br><br>[1] Adapted from The Patient and Public Engagement Evaluation Tool. "The Patient and Public Engagement Evaluation Tool has been licensed under a Creative Commons Attribution-NonCommercial-Share Alike 4.0 International License. © 2015, Julia Abelson and the PPEET Research-Practice Collaborative. McMaster University. All rights reserved."	descriptive
61	participant_name3	Please enter your first and last name:	text, Required, Identifier

62	content6mo_willingness	Section Header: *INSTRUCTIONS This questionnaire has several statements and you can indicate the extent to which you agree with each of them by selecting the appropriate box.*<br>The information increased my willingness to influence policy for children with disabilities	radio (Matrix)<br>0 Strongly disagree<br>1 Disagree<br>2 Neither agree nor disagree<br>3 Agree<br>4 Strongly agree
63	content6mo_emotion	The information in the policy briefs affected me emotionally.	radio (Matrix)<br>0 Strongly disagree<br>1 Disagree<br>2 Neither agree nor disagree<br>3 Agree<br>4 Strongly agree
64	content6mo_understand	The information in the policy briefs is easy to understand.	radio (Matrix)<br>0 Strongly disagree<br>1 Disagree<br>2 Neither agree nor disagree<br>3 Agree<br>4 Strongly agree
65	usebrief_yn	Have you been able to use the information from the policy briefs and the dialogue?	yesno<br>1 Yes<br>0 No<br>Custom alignment: LH
66	usebrief_why<br>Show the field ONLY if:<br>[usebrief_yn] = '1'	Why?	notes<br>Custom alignment: LH
67	usebrief_whynot<br>Show the field ONLY if:<br>[usebrief_yn] = '0'	Why not?	notes<br>Custom alignment: LH
68	usebrief_example<br>Show the field ONLY if:<br>[usebrief_yn] = '1'	Can you give a concrete example of how you have used the information?	notes<br>Custom alignment: LH
69	usebrief_factors	What factors have most strongly affected your ability to use or not use this information?	notes<br>Custom alignment: LH
70	usebrief_factors_examples	What else would have been helpful for you to use/apply the information? Why?	notes<br>Custom alignment: LH
71	usebrief_future_use	Do you expect to use the information acquired during the meeting and through the briefs in the future? If yes, how? If no, why?	notes<br>Custom alignment: LH
72	postdialogues_6mo_complete	Section Header: *Form Status*<br>Complete?	dropdown<br>0 Incomplete<br>1 Unverified<br>2 Complete
Instrument: **post-dialogues_12mo** (postdialogues_12mo)			
73	instructions_v2_at12mo	It has been 12 months since you participated in the BC Policy Dialogue in childhood disabilities.<br><br>Now we would like to understand if the information that you received during the Dialogue and through the briefs was used somehow or applied in any way, and if not, the reasons why.<br><br>All information you provide will be anonymous and confidential.<br><br>This survey [1] should take only ten minutes of your time.<br><br>[1] Adapted from The Patient and Public Engagement Evaluation Tool. "The Patient and Public Engagement Evaluation Tool has been licensed under a Creative Commons Attribution-NonCommercial-Share Alike 4.0 International License. © 2015, Julia Abelson and the PPEET Research-Practice Collaborative. McMaster University. All rights reserved."	descriptive
74	participant_name3_at12mo	Please enter your first and last name:	text, Required, Identifier
75	content6mo_willingness_at12mo	Section Header: *INSTRUCTIONS This questionnaire has several statements and you can indicate the extent to which you agree with each of them by selecting the appropriate box.*<br>The information increased my willingness to influence policy for children with disabilities	radio (Matrix)<br>0 Strongly disagree<br>1 Disagree<br>2 Neither agree nor disagree<br>3 Agree<br>4 Strongly agree
76	content6mo_emotion_at12mo	The information in the policy briefs affected me emotionally.	radio (Matrix)<br>0 Strongly disagree<br>1 Disagree<br>2 Neither agree nor disagree<br>3 Agree<br>4 Strongly agree
77	content6mo_understand_at12mo	The information in the policy briefs is easy to understand.	radio (Matrix)<br>0 Strongly disagree<br>1 Disagree<br>2 Neither agree nor disagree<br>3 Agree<br>4 Strongly agree

78	usebrief_yn_at12mo	Have you been able to use the information from the policy briefs and the dialogue?	yesno<br>1 Yes<br>0 No<br><br>Custom alignment: LH
79	usebrief_why_at12mo<br><br>Show the field ONLY if:<br>[usebrief_yn_at12mo] = '1'	Why?	notes<br>Custom alignment: LH
80	usebrief_whynot_at12mo<br><br>Show the field ONLY if:<br>[usebrief_yn_at12mo] = '0'	Why not?	notes<br>Custom alignment: LH
81	usebrief_example_at12mo<br><br>Show the field ONLY if:<br>[usebrief_yn_at12mo] = '1'	Can you give a concrete example of how you have used the information?	notes<br>Custom alignment: LH
82	usebrief_factors_at12mo	What factors have most strongly affected your ability to use or not use this information?	notes<br>Custom alignment: LH
83	usebrief_factors_examples_at12mo	What else would have been helpful for you to use/apply the information? Why?	notes<br>Custom alignment: LH
84	usebrief_future_use_at12mo	Do you expect to use the information acquired during the meeting and through the briefs in the future? If yes, how? If no, why?	notes<br>Custom alignment: LH
85	postdialogues_12mo_complete	Section Header: *Form Status*<br>Complete?	dropdown<br>0 Incomplete<br>1 Unverified<br>2 Complete
